# Mediastinal Lymphangioma of the Adult: Case Report and Literature Review

**DOI:** 10.3390/clinpract16080144

**Published:** 2026-08-06

**Authors:** Mihaela-Beatrice Tivadar, Amir-Andrei Sabha, Vasile Grigorie, Raluca Bobocea, Andrei-Cristian Bobocea

**Affiliations:** “Marius Nasta” Institute of Pneumology, 050159 Bucharest, Romania; tivadarbeatrice@gmail.com (M.-B.T.); vasile.grigorie@gmail.com (V.G.); raluca84@gmail.com (R.B.); andrei.bobocea@gmail.com (A.-C.B.)

**Keywords:** mediastinal lymphangioma, cystic hygroma, costophrenic recess, magnetic resonance imaging, thoracotomy

## Abstract

**Introduction**: Mediastinal lymphangiomas are rare, benign vascular malformations of the lymphatic system that are seldom diagnosed in adults. Due to their varied clinical presentation and heterogeneous imaging characteristics, they pose a significant preoperative diagnostic challenge, frequently mimicking other mediastinal masses. **Materials and Methods**: We conducted a thorough search of the PubMed/MEDLINE, PubMed Central, and Google Scholar databases. We included articles from 2000 to 2026. Our search yielded 150 articles, out of which we selected 45 articles. Inclusion criteria were strictly limited to adult patients with a solitary mediastinal lymphangioma. Cases involving diffuse systemic lymphangiomatosis, mixed histological features such as lymphangiomyomas or hemangiolymphangioma or studies on pediatric populations were explicitly excluded from the analysis. We also reported a case of mediastinal lymphangioma in a 42-year-old patient, which was surgically resected at the “Marius Nasta” National Institute of Pneumology in Bucharest, Romania. **Case Description**: An asymptomatic 42-year-old male presenting with exertional chest discomfort following minor trauma demonstrated a lower left hemithorax opacity on chest X-ray. Imagistic studies revealed a 13 × 9 × 6 cm cystic mass in the left supradiaphragmatic costophrenic recess. The patient underwent a complete radical excision. The postoperative recovery was uneventful. Histopathology confirmed a benign cystic mediastinal lymphangioma. **Conclusions**: Adult mediastinal lymphangiomas are rare entities that require a high index of clinical suspicion. While advanced cross-sectional imaging is invaluable for delineating their characteristics, definitive diagnosis relies on histopathological confirmation. Complete surgical resection, increasingly performed via minimally invasive approaches or standard thoracotomy, remains the therapeutic gold standard, offering an excellent long-term prognosis with virtually no risk of recurrence. For inoperable cases, several non-surgical approaches are available.

## 1. Introduction

Described initially by Redenbacker in 1828 and initially named “cystic hygroma” [1], mediastinal lymphangiomas (LMs) are rare, benign vascular malformations characterized by the abnormal proliferation of lymphatic vessels [2,3], which represent 0.7–4.5% of all mediastinal tumors in adults [4]. They are predominantly considered congenital anomalies resulting from the failure of embryonal lymphatic spaces to establish functional communications with the main lymphatic system. Consequently, the vast majority of cases—approximately 90%—manifest in early childhood, typically diagnosed before the age of 2, with a marked predilection for the cervical and axillary regions [5,6].

When located within the thoracic cavity, they are most frequently discovered in the anterior and superior mediastinal compartments, while localization in the posterior mediastinum or the diaphragmatic recesses is exceptionally uncommon [7]. Because these tumors are slow-growing and histologically benign, they often remain clinically silent for decades. When symptoms do arise, they are typically insidious and non-specific, stemming from the gradual compression or displacement of adjacent intrathoracic structures, resulting in cough, dyspnea, or vague chest discomfort [8].

Preoperative diagnosis in adult patients presents a formidable clinical challenge. On cross-sectional imaging, LM can display highly heterogeneous characteristics frequently mimicking other mediastinal lesions such as thymomas, bronchogenic cysts, teratomas, lipomas, or even low-grade sarcomas. While advanced imaging modalities, particularly magnetic resonance imaging (MRI), offer superior soft tissue contrast essential for mapping the internal architecture of these complex lesions [4,9,10,11,12], definitive diagnosis remains strictly histopathological [8].

Currently, complete surgical excision (either by thoracotomy or video-assisted thoracic surgery—VATS) represents the therapeutic gold standard to prevent local mass-effect complications and avoid the risk of recurrence [13,14]. However, in asymptomatic cases or in cases of inoperability, conservative methods may be used [15,16,17,18,19,20].

Here, we present a rare clinical case of a 13 × 9 × 6 cm, multiloculated supradiaphragmatic mediastinal lymphangioma in a 42-year-old male, successfully managed via radical surgical resection through a standard left thoracotomy at the ‘Marius Nasta’ National Institute of Pneumology.

Additionally, we perform a comprehensive review of the literature on adult solitary mediastinal lymphangiomas published between 2000 and 2026 to evaluate clinical presentations, diagnostic approaches, and surgical outcomes.

## 2. Materials and Methods

### 2.1. Case Report Presentation

We report a rare clinical case of a large, multiloculated supradiaphragmatic mediastinal lymphangioma in a 42-year-old male. The patient was evaluated, managed, and successfully treated with radical surgical resection via a standard left thoracotomy at the “Marius Nasta” National Institute of Pneumology in Bucharest, Romania. Clinical history, diagnostic imaging (chest CT), surgical findings, histopathological analysis, and postoperative follow-up data were collected and documented.

### 2.2. Literature Search Strategy

We conducted a thorough search of the PubMed/MEDLINE, PubMed Central, and Google Scholar databases. A comprehensive literature search was performed utilizing combinations of the following medical subject headings and keywords: [(“lymphangioma” OR “mediastinal lymphangioma” OR “cavernous lymphangiomas” OR “cystic hygromas” OR “capillary lymphangioma” OR “lymphangioma simplex”) AND (“chylothorax” OR “chylous pleural effusion” OR “chylous effusion” OR “chylous lung”)]. We included articles from 2000 to 2026. Our search yielded 150 articles in total, out of which we selected 45 articles for inclusion. Inclusion criteria were strictly limited to adult patients presenting with a solitary mediastinal lymphangioma. Cases involving diffuse systemic lymphangiomatosis, mixed histological features such as lymphangiomyomas or hemangiolymphangioma or pediatric populations were explicitly excluded from the analysis.

### 2.3. Ethical Considerations

Written informed consent was obtained from the patient prior to publication for the report of his clinical case and the use of any accompanying diagnostic and surgical images. All personal patient identification data were fully anonymized to protect patient confidentiality. This study was conducted in accordance with the ethical principles of the Declaration of Helsinki and was reviewed and approved by the Ethics Committee of the “Marius Nasta” National Institute of Pneumology (protocol code 9164—19 January 2026).

### 2.4. Statistical Analysis

Descriptive statistics were used to summarize clinical features, demographic data, tumor dimensions, surgical approaches, and outcomes extracted from the included literature. Continuous variables were presented as mean ± standard deviation (SD) or median with interquartile range (IQR), as appropriate based on data distribution. Categorical variables were expressed as absolute counts and percentages (*n*%). All data processing and statistical calculations were conducted using Microsoft Excel.

## 3. Results

### 3.1. Case Report

We present a clinical case of LM treated at the “Marius Nasta” National Institute of Pneumology in Bucharest. Written informed consent was obtained from the patient for publication of this case report and any accompanying images.

The patient was a 42-year-old male who presented with mild, nonspecific chest discomfort that typically occurred after moderate-to-high physical exertion. Additionally, the patient had a history of minor chest trauma from a road traffic accident several years prior, which did not require hospitalization. Upon physical examination, the patient was asymptomatic and stable.

**Figure 1:**
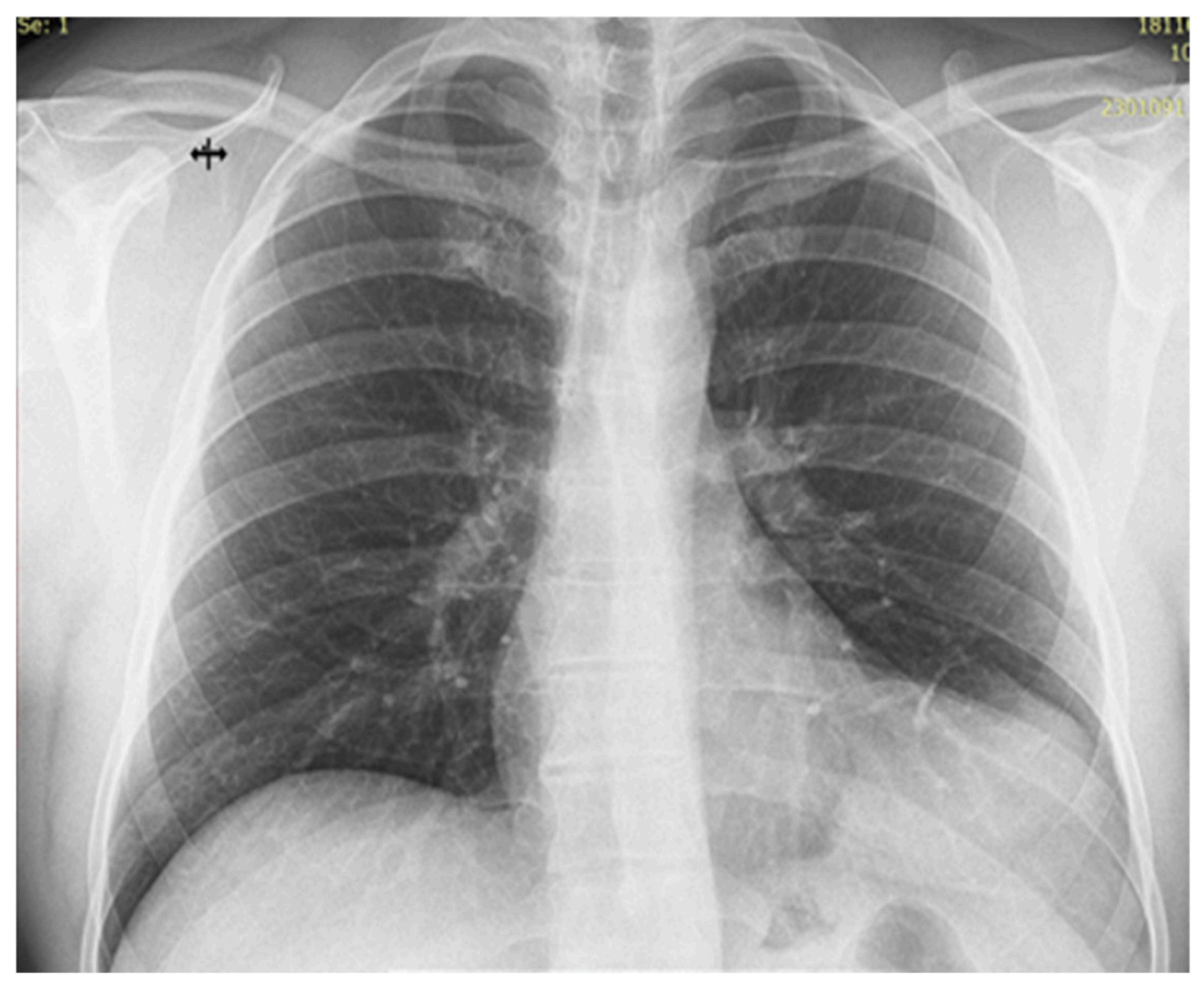
Initial posteroanterior chest radiograph demonstrating a well-defined opacity in the lower left hemithorax.

**Figure 2:**
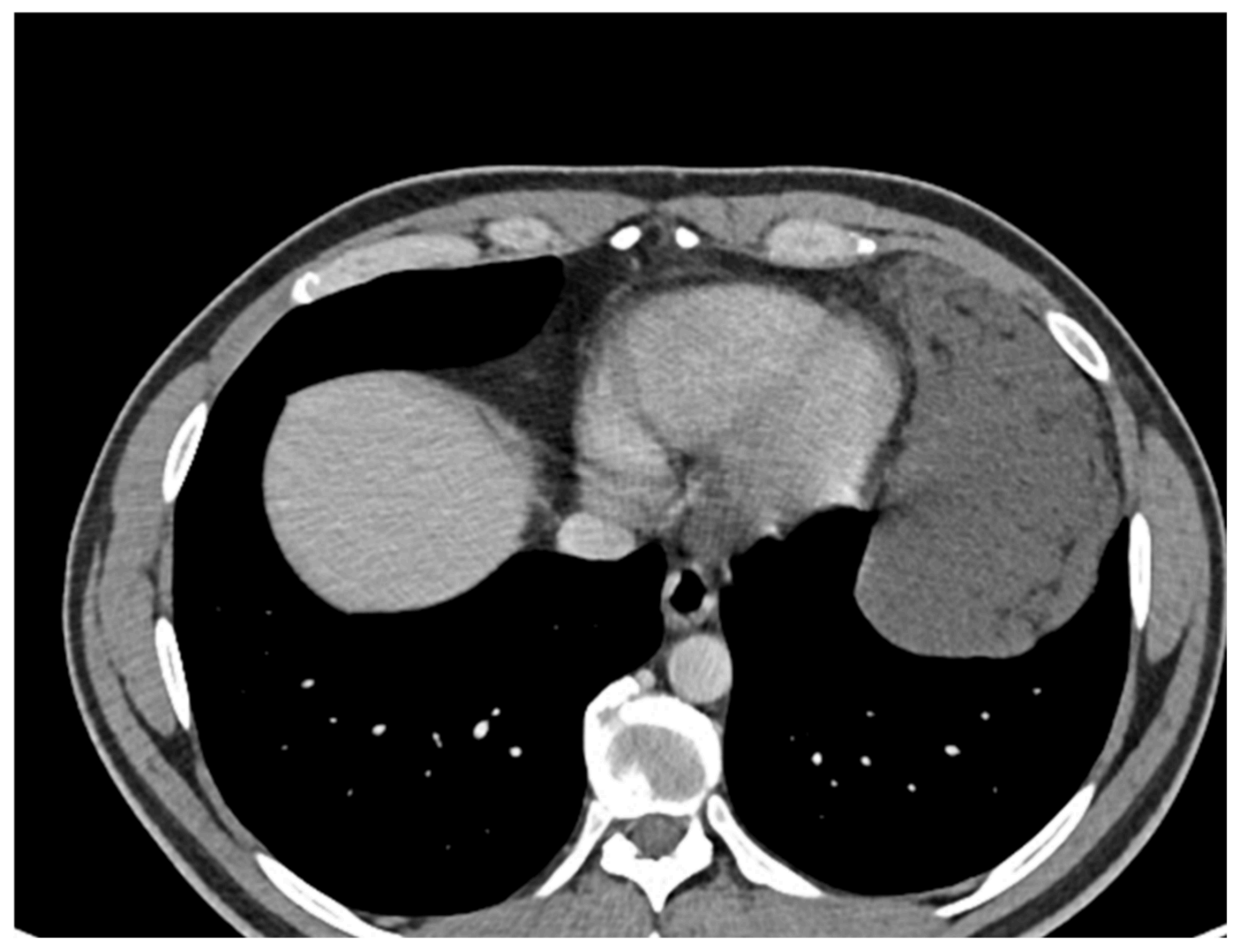
Axial CT scan displaying a heterogeneous 100×65 mm mass with fluid and adipose attenuation components in the left supradiaphragmatic costophrenic recess.

The chest X-ray (Figure 1) revealed an opacity in the lower part of the left hemithorax. A subsequent computed tomography (CT) examination revealed a 100 × 65 mm mass in the left costophrenic recess. The mass exhibited mixed components, including adipose tissue as well as fluid and para-fluid densities, without a clear demarcation from the diaphragm (Figure 2).

**Figure 3:**
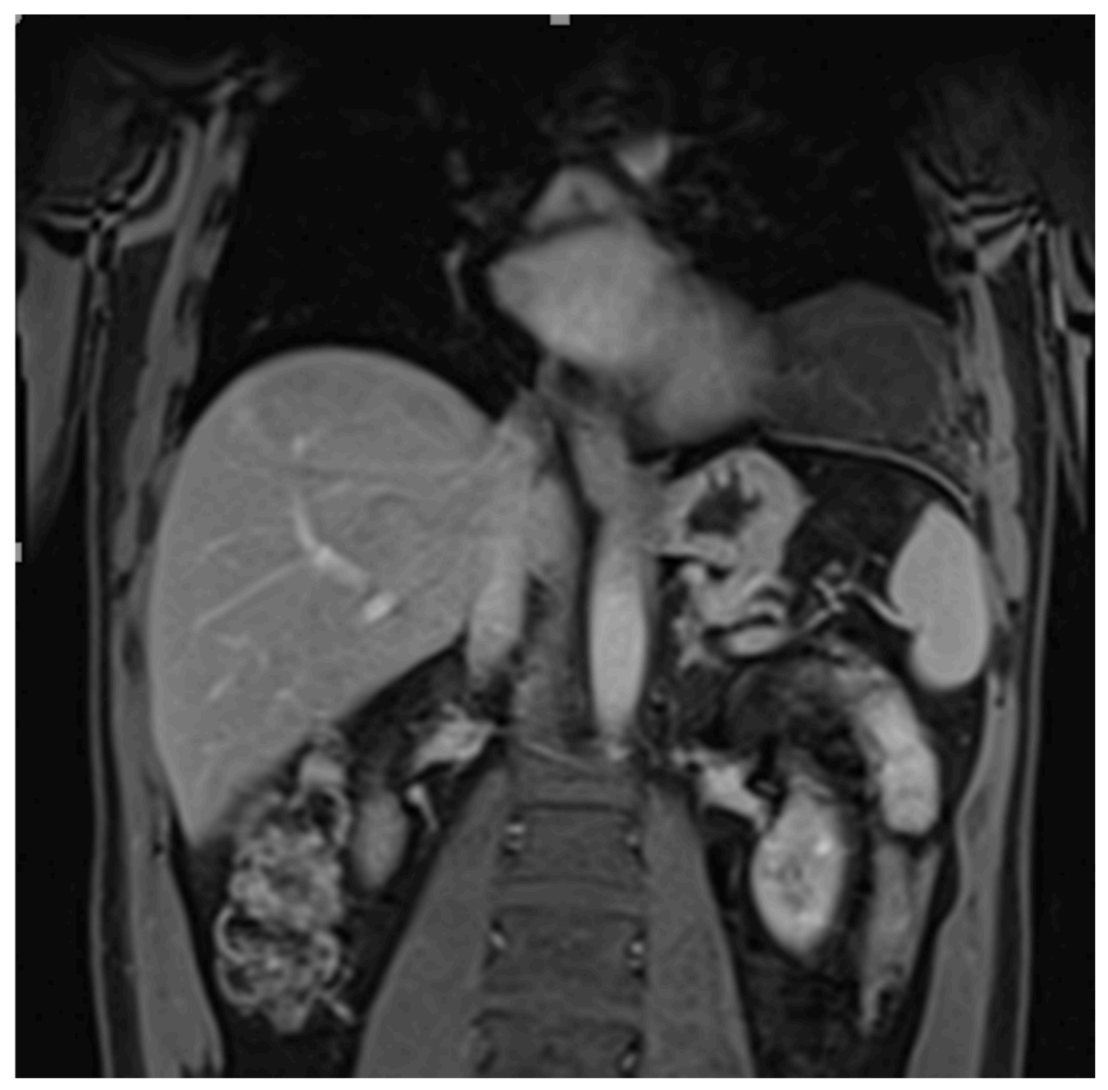
Coronal T2-weighted magnetic resonance imaging (MRI) view confirming a multiloculated, septated cystic lesion (13×9×6 cm) with hyperintense fluid content at the left supradiaphragmatic level.

To further elucidate the character and tissue composition of the mass, a chest MRI was performed. The scan revealed a well-defined, multiloculated cystic lesion characterized by internal septations and a complex mucoid-proteinaceous component. Measuring 13 × 9 × 6 cm, the mass demonstrated a distinct micronodular architecture within its anterior aspect at the left supradiaphragmatic level, notably without invading or distorting the anatomy of the adjacent structures. Based on these advanced imaging characteristics, a presumptive diagnosis of mediastinal lymphangioma was established (Figure 3).

Pulmonary function testing, including spirometry, demonstrated normal lung volumes, as well as preserved diffusing capacity for carbon monoxide (DLCO) and transfer coefficient (KCO) values. Routine laboratory evaluations were unremarkable, yielding results strictly within normal physiological parameters, with the exception of a marginal elevation in serum cholesterol and triglycerides.

Given the diagnostic ambiguity preoperatively, a broad differential diagnosis was considered:

Thymoma, secondary to imaging findings suggesting an anterior mediastinal origin;

Diaphragmatic hernia, raised as a strong possibility in light of the patient’s prior history of chest trauma;

• Lipoma or liposarcoma, hypothesized due to the heterogeneous tissue densities and complex internal composition observed on cross-sectional imaging;
• Lymphangioma, which remained the primary presumptive diagnosis supported by the characteristic MRI findings.

**Figure 4:**
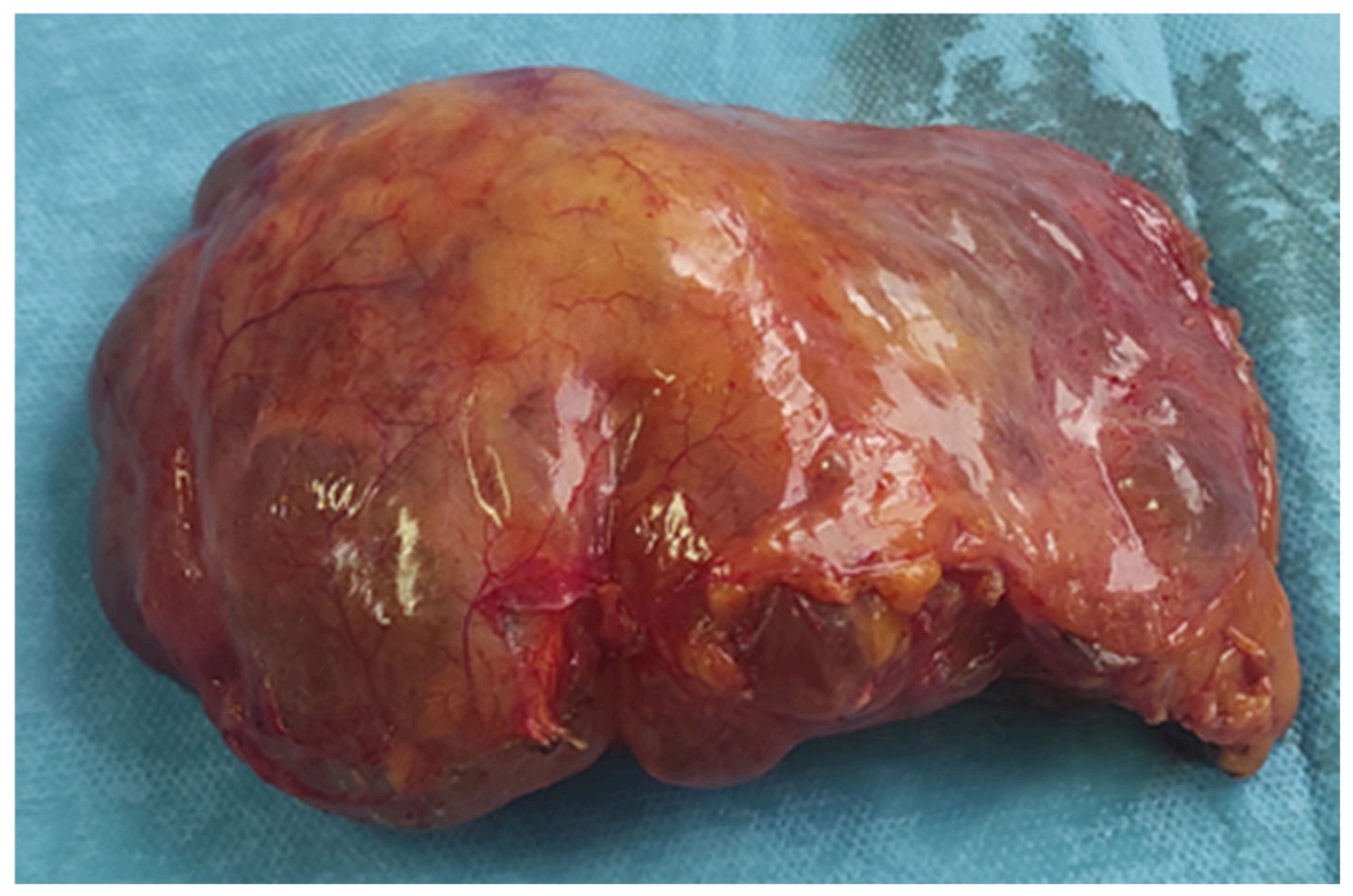
Gross pathological specimen following complete surgical resection via left anterolateral thoracotomy, showing a well-encapsulated multiseptated cystic mass.

The patient underwent definitive surgical resection via a left anterolateral thoracotomy through the fifth intercostal space. Intraoperatively, a well-encapsulated, distinct mass was identified, originating from the anterior mediastinum and extending into the left intrapleural space, specifically occupying the cardiophrenic recess. Only minimal, easily dissectible adhesions to the lingula were noted (Figure 4).

Complete radical excision of the tumor was achieved without any intraoperative incidents or complications. The postoperative course was uneventful, and the patient was discharged in stable condition on postoperative day 3.

**Figure 5:**
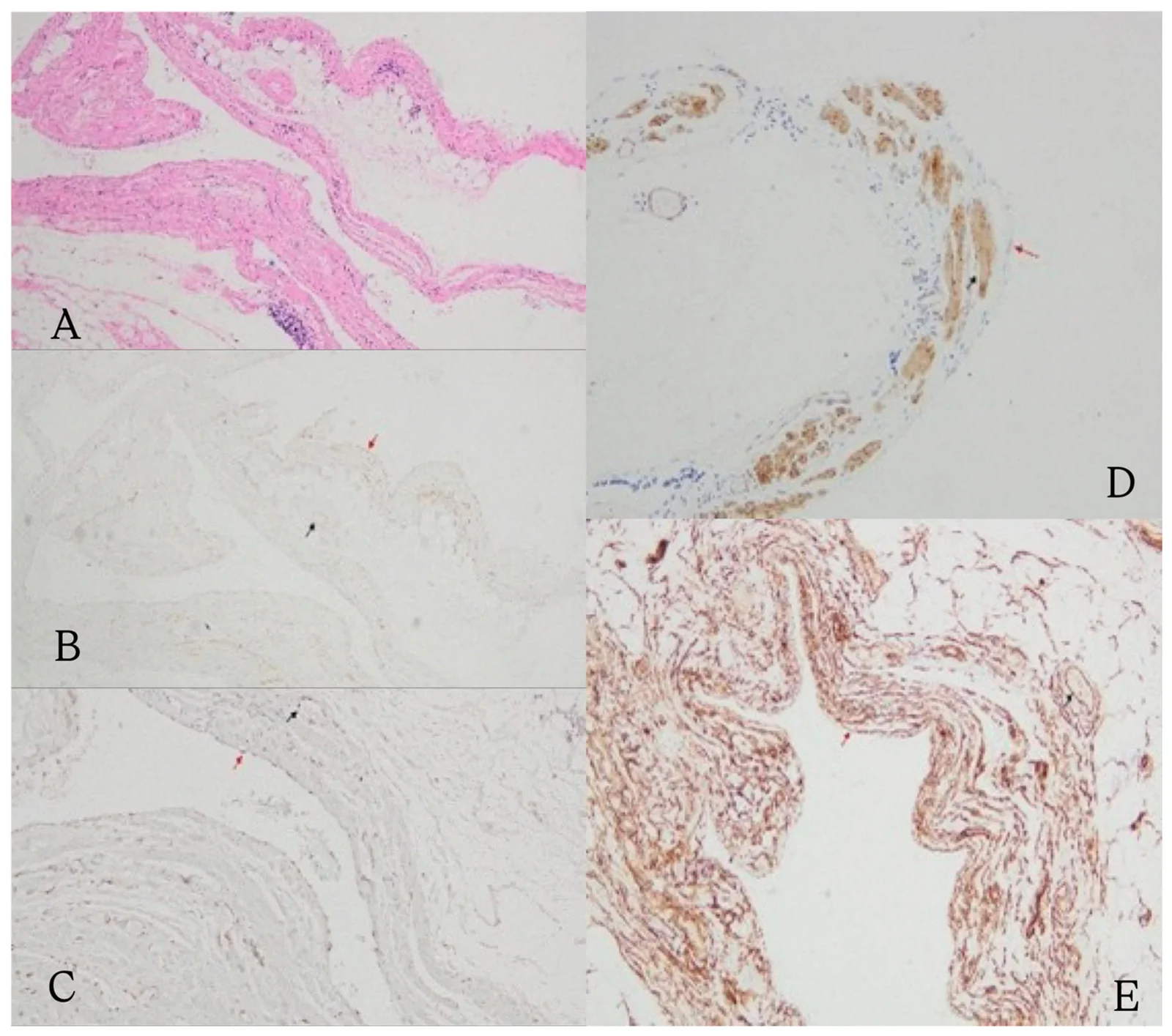
Histopathological and immunohistochemical features of the resected mediastinal lymphangioma: (**A**) Hematoxylin and eosin (HE) staining demonstrating dilated cystic spaces lined by attenuated endothelial cells with fibrous walls; (**B**) CD31 positivity (40×) in endothelial cells lining the lymphatic channels and internal capillary control; (**C**) ERG nuclear expression (100×) confirming endothelial lineage; (**D**) smooth muscle actin (SMA, 100×) highlighting smooth muscle bundles within the cyst wall (black arrow) while endothelial lining remains negative (red arrow); (**E**) CD34 endothelial positivity (100×).

The diagnosis of mediastinal lymphangioma was confirmed through histopathological and immunohistochemical analysis which showed positive CD31 and CD34 (Figure 5).

### 3.2. Literature Review

A comprehensive review of the indexed literature from 2000 to the present highlights the clinical heterogeneity and diagnostic challenges associated with LM. After the exclusion criteria were applied, 45 publications were found, reporting 67 cases of LM of the adult (Table A1).

In the reported cases, ages ranged from 18 to 77 years, with a mean age of 45 years. There were 35 male patients and 32 female patients.

There were 28 asymptomatic patients (41.8%) and 39 symptomatic patients (58.2%). The symptoms are described in Table 1.

The main diagnostic tool was CT for 63 patients (94%) and MRI was used for 17 patients (25.37%) either to better describe the LM identified by CT, or as main investigation (2 patients—3%). US was used for three patients (4.47%), in order to guide fluid aspiration. Other diagnostic methods were fibrobronchoscopy in seven cases (10.44%) and fine needle aspiration for four patients (5.97%).

Most LMs were positioned in more than 2 mediastinal compartments, 2 (3%) occupied the entire mediastinum, 8 (11.94%) were positioned strictly in the superior mediastinum, 18 (26.86%) in the anterior mediastinum, while 6 (8.95%) were in the middle mediastinum and 4 (5.97%) in the posterior mediastinum.

Regarding the size of the LM, data were available for 38 patients, sizes were between 2 and 22 cm, the mean size was 8.4 cm (±4.3 cm), median size was 7.5 cm, and 44.7% of the cysts ranged from 5 to 10 cm.

Fifty patients (74.62%) underwent surgery. Table 2 summarizes the specific types of approaches.

For six patients mediastinoscopy was used, mostly for biopsies, with only one case where resection of a small LM was performed by this approach (Table 3).

A non-surgical approach was chosen in 17 cases (25.4%), primarily due to extensive infiltration, high perioperative risk, or underlying systemic disease (Table 4).

Postoperative complications included two cases of prolonged lymphatic drainage and one case of transient nerve palsies secondary to difficult dissections [32].

In the 50 cases treated surgically, recurrence was reported in 4 patients; the rest of the publications reported no recurrence.

## 4. Discussion

LMs are exceptionally rare benign vascular malformations, accounting for less than 1% of all mediastinal masses. Derived from sequestrations of lymphatic tissue that fail to establish normal connections with the lymphatic system, these slow-growing lesions are predominantly diagnosed in early childhood, making adult presentations particularly unusual [8,32,33].

The literature review demonstrates that, while this pathology can present at any stage of life, with sufficient cases reported in infants and young children, when diagnosed at an adult age it is more frequently diagnosed in middle-aged adults [8]. In the reported cases ages spanned from 18 to 77, the average age being 45. There seems to be no exclusive predilection for either gender, though males appear slightly more prominent.

In a span of 20 years, our literature review comprises 45 publications and 67 cases, which show the rarity of this pathology, hence, the difficulty in making a correct differential diagnosis if we do not take into account rare entities such as LM.

In our report, we presented a 42-year-old male who developed non-specific exertional chest discomfort, with a clinical background of minor chest trauma several years prior. Differential diagnosis was initially oriented towards more frequent pathologies which would have explained these non-specific symptoms.

In our literature review of 67 adult primary LM cases, clinical presentations varied widely: 41.8% of patients were completely asymptomatic—with their lesions discovered incidentally during routine radiography—while 58.2% presented with symptomatic mass-effect complaints. Among symptomatic individuals across the literature, the most frequent manifestations were exertional dyspnea (19.4%), vague chest discomfort (13.4%), and chronic dry cough (11.9%), with rare emergency presentations such as cardiac tamponade (3.0%) or superior vena cava syndrome (1.5%) resulting from severe local compression. In comparison, our 42-year-old male patient presented with mild, non-specific exertional chest discomfort that aligned with the most common symptomatic complaints reported in the literature. However, a distinct feature of our case was the patient’s history of minor chest trauma from a road traffic accident several years prior. Combined with the tumor’s unusual supradiaphragmatic costophrenic recess localization and mixed attenuation on CT, this traumatic history initially raised strong suspicion for a post-traumatic diaphragmatic hernia—a diagnostic ambiguity rarely described in the reviewed literature, where most incidental or symptomatic presentations trigger a differential diagnosis limited to primary mediastinal cysts or thymic neoplasms.

Initial chest radiography identified a lower left hemithorax opacity (Figure 1). Non-contrast and contrast-enhanced chest CT revealed a 100 × 65 mm mass in the left costophrenic recess with mixed fluid, soft-tissue, and adipose-like attenuation. Subsequent chest MRI confirmed a 13 × 9 × 6 cm multiloculated, septated cystic mass exhibiting characteristic hyperintense T2 fluid content and micronodular septal architecture.

A notable diagnostic difficulty in our case was the presence of mixed fat-and-fluid attenuation on the initial CT scan. While LMs classically exhibit uniform water-attenuation (0–20 HU), proteinaceous debris, intracystic hemorrhage, or trapped epicardial/pericardial fat within internal septa can create heterogeneous tissue densities [10,11,34]. In our case, this attenuation pattern, combined with the history of chest trauma, created a broad differential diagnosis including:

• Diaphragmatic hernia: suspected due to trauma history and inferior supradiaphragmatic positioning.
• Lipoma or liposarcoma: considered because of adipose-density tissue elements on CT.
• Thymoma or bronchogenic cyst: typical primary mediastinal cystic differential entities.

This diagnostic challenge highlights the indispensable role of T2-weighted MRI. MRI delineated the multiloculated, septated, mucoid-proteinaceous fluid content and micronodular wall architecture, correctly shifting the primary diagnostic hypothesis to LM prior to surgery. It is usually the diagnostic method of choice when CT results are unclear [12].

In our comprehensive literature review, LMs demonstrated a clear anatomical predilection for the superior and anterior mediastinal compartments (over 38% of isolated anterior/superior cases, with many spanning multiple compartments). Localization within the posterior mediastinum (5.97%) or middle mediastinum (8.95%) is far less frequent. Our patient’s tumor was localized in the left supradiaphragmatic costophrenic/cardiophrenic recess. LM in this specific inferior recess is unusual. They likely originate from ectopic diaphragmatic or lower anterior mediastinal lymphatic tissue, requiring careful intraoperative isolation from both the phrenic nerve and the diaphragmatic surface.

Across the published cases where dimensions were recorded (*N* = 38), the mean maximum tumor diameter was 8.4 ± 4.3 cm (median 7.5 cm), with nearly half of the cohort (45.8%) presenting with masses between 5 and 10 cm. Measuring 13 × 9 × 6 cm, our patient’s mass falls into the upper tier of “giant” mediastinal lymphangiomas (>10 cm). Giant lesions in adults pose an increased risk of compression-induced complications—such as superior vena cava syndrome, cardiac tamponade, or tracheal displacement—as well as intraoperative rupture [14,29,35].

Regarding other diagnostic tools, the review showed that ultrasonography (US) and Color Doppler imaging were successfully utilized in a few reported cases [36,37], particularly for lesions extending into the supraclavicular or upper abdominal regions. Although US is limited by the acoustic barriers of the bony thoracic cage, it remains an excellent, non-invasive, cost-effective tool for confirming the purely cystic, fluid-filled nature of accessible masses and evaluating real-time vascularity or blood flow absence within the cystic spaces, and it can also aid in guiding a percutaneous fluid aspiration or biopsy [28,38]. Furthermore, traditional or advanced lymphography has occasionally been employed to definitively map out the direct connections or lack thereof between the mediastinal mass and the central lymphatic pathways, such as the thoracic duct [30]. Finally, positron emission tomography–computed tomography (PET-CT) was incorporated in more recent complex presentations to rule out malignancy. Because benign lymphangiomas characteristically demonstrate a lack of metabolic activity, PET-CT can play a crucial role in the differential diagnosis, helping clinicians confidently differentiate these masses from highly metabolic, aggressive mediastinal malignancies, sarcomas, or lymphoma [8,24].

Biopsy was performed in the only case of esophageal LM found in this series, in which the extent of the tumor and adherence to regional structures made it impossible to resect. The lack of severe digestive symptoms was also a factor in choosing the therapeutic approach [39]. In our case biopsy was not considered since the MRI oriented the diagnosis towards a resectable LM, and thus histopathological diagnosis was obtained from the surgically resected specimen (Figure 4).

Histopathological and immunohistochemical evaluations confirmed a benign cystic mediastinal lymphangioma, with strong positivity for vascular endothelial markers CD31 and CD34. The patient’s postoperative course was uneventful, and long-term follow-up demonstrated no evidence of recurrence (Figure 5).

Our comprehensive review highlights a major technological shift over the past two decades: 48.0% of surgically treated patients underwent minimally invasive procedures (VATS or RATS), while 52.0% were managed via open approaches (thoracotomy or median sternotomy). VATS and RATS demonstrate excellent efficacy, reduced postoperative morbidity, and shortened hospital stays [14,40,41,42] and are excellent for lesions under 5–8 cm. In our case, we consider that open anterolateral thoracotomy remains a critical and safe approach for giant (>10 cm) supradiaphragmatic LMs. Open access allowed complete en bloc excision, prevention of intraoperative cyst rupture, and precise mobilization from the pericardium and lingula without converting from a compromised minimally invasive field.

Postoperative complications remain exceedingly rare, with isolated reports of prolonged lymphatic drainage [25,43] or transient nerve palsies secondary to difficult dissections [32].

Ultimately, when complete margin-negative surgical resection is achieved, the long-term prognosis is excellent, and recurrence is exceedingly rare, validating radical excision as the therapeutic gold standard. The only study that reported recurrences was Park et al. (2006) [22] describing five recurrences in four patients (33%). The mean time to primary recurrence from the time of initial surgery was 3.6 years, and a single additional recurrence from the time of initial surgery was noted at 10.0 years [22] showing a follow-up of 25 years which is singular in this series.

Histopathological confirmation identified these lesions as benign cystic or cavernous lymphangiomas, with the overall majority being cystic lymphangiomas. Definite confirmation is often obtained by immunohistochemical analysis. Positive cellular expression of vascular endothelial markers (CD31, CD34, and ERG) confirms the definitive lymphatic-endothelial lineage of the cells lining the cystic spaces. Smooth muscle actin (SMA) staining shows the presence of bundle-like smooth muscle fibers confined within the cystic walls, aiding pathologists in sub-typing the lesion (e.g., separating cystic variants from smooth muscle-rich lymphangiomyomas) [44].

While radical surgical excision remains the definitive standard of care for adult LM, non-surgical interventions have emerged as crucial alternatives for patients who are poor surgical candidates, present with extensive infiltrating lesions, or experience post-resection recurrences. Currently, image-guided percutaneous sclerotherapy—utilizing sclerosants such as OK-432 (Picibanil), bleomycin, doxycycline, or ethanol—serves as the primary non-surgical modality, triggering an intense local inflammatory reaction that obliterates the lymphatic endothelial lining and causes cyst collapse [17,45,46,47]. However, sclerotherapy displays variable efficacy in mediastinal lymphangiomas, working exceptionally well for isolated macrocystic lesions but demonstrating limited success in microcystic or polycystic, multiloculated variants. Looking to the future, the therapeutic landscape is shifting toward novel targeted pharmacotherapies informed by molecular biology. These alternatives were typically reserved for two distinct clinical cohorts: patients presenting acutely with cardiopulmonary or compressive symptomatology, or conversely, entirely asymptomatic individuals in whom the technical risks of invasive thoracic intervention significantly outweighed the potential benefits of prophylactic surgical removal.

One last aspect that resulted from our literature review is the possibility that LM develops in the context of a systemic disease or syndromic associations. A striking example is the coexistence of lymphatic proliferation with Gorham–Stout disease (“vanishing bone disease”), where benign lymphatic hyperplasia aggressively infiltrates osseous structures, leading to progressive osteolysis of adjacent bones, such as the clavicle or ribs [25]. Additionally, a rare association with tuberous sclerosis complex has been documented [48], underscoring how multi-system genetic mutations can occasionally trigger anomalous thoracic lymphatic developments. Recognizing rare phenotypes is clinically vital, as they necessitate a comprehensive, multidisciplinary workup extending far beyond standard localized thoracic management [25,48,49].

This study has a few limitations. First, the literature review is limited by publication bias, as smaller or uncomplicated cases of adult mediastinal lymphangioma may be underreported. Second, due to the rarity of the condition, our review relies on heterogeneous case reports and small series. Finally, long-term follow-up data across published cases vary significantly, limiting conclusions on multi-decade recurrence rates.

Given the rarity and surgical complexity of adult LM, future prospective multi-center registries are needed to establish standardized diagnostic and therapeutic algorithms. Comparative studies evaluating the feasibility, safety, and outcomes of minimally invasive techniques (VATS and robotic platforms) versus traditional open thoracotomy for giant (>8 cm) lesions are warranted. The discovery of somatic activating mutations in the PIK3CA (Phosphatidylinositol-4,5-Bisphosphate 3-Kinase Catalytic Subunit Alpha) gene across various lymphatic malformations has driven the adoption of biological agents, most notably the oral mammalian target of rapamycin (mTOR) inhibitor sirolimus (rapamycin). Sirolimus directly inhibits vascular endothelial growth factor (VEGF) signaling and downstream lymphangiogenesis, demonstrating remarkable efficacy in shrinking complex, refractory lymphatic anomalies [19]. Future development hinges on refining these targeted systemic therapies—including second-generation PI3K/mTOR pathway inhibitors—and evaluating them in formal adult clinical trials, either as primary non-invasive medical treatments or as neoadjuvant cytoreductive therapies to downsize giant mediastinal masses prior to minimally invasive surgical resection.

In conclusion, radical complete surgical excision remains the cornerstone of management, offering an excellent long-term prognosis and minimal recurrence risk when clear surgical margins are achieved [33].

## 5. Conclusions

Adult LMs are rare, benign vascular malformations that present a significant preoperative diagnostic challenge due to their heterogeneous, multi-septated cystic architecture, which frequently mimics other mediastinal masses. While 41.8% of patients are asymptomatic, localized mass effects can cause insidious respiratory symptoms or severe emergencies like cardiac tamponade. Advanced cross-sectional imaging, particularly MRI, is essential for mapping these non-invasive, fluid-filled lesions, though definitive diagnosis requires histopathological confirmation. Radical surgical excision remains the therapeutic gold standard to prevent local recurrence, with contemporary clinical practice successfully shifting toward minimally invasive approaches like VATS and RATS to optimize patient recovery.

## Figures and Tables

**Table 1:** Clinical presentation of reported adult mediastinal lymphangioma cases (N=67).

Clinical Presentation/Symptom Category	No. of Patients (*n*)	Percentage (%)
Asymptomatic (Incidental Discovery)	28	41.8%
Symptomatic	39	58.2%
1. Respiratory and Cardiovascular Compression		
— Dyspnea on Exertion	13	19.4%
— Chronic Dry Cough	8	11.9%
— Palpitations/Arrhythmias	3	4.5%
2. Thoracic Pain and Discomfort		
— Vague Chest Discomfort	9	13.4%
— Acute/Pleuritic Chest Pain	6	9.0%
3. Severe Mass Effect and Emergencies		
— Cardiac Compression	2	3.0%
— Superior Vena Cava Syndrome	2	3.0%

**Table 2:** Surgical approaches in adult mediastinal lymphangioma management (N=50).

Approach Category	Specific Surgical Method	Percentage (*n*/*N*)
Minimally Invasive	VATS/RATS (Total)	48.0% (n=24)
	Completed minimally invasive (VATS/RATS)	42.0% (n=21)
	Converted to open thoracotomy	6.0% (n=3)
Open Approach	Thoracotomy/Sternotomy (Total)	52.0% (n=26)
	Thoracotomy	36.0% (n=18)
	Median sternotomy	16.0% (n=8)

Abbreviations: VATS = video-assisted thoracic surgery; RATS = robotic-assisted thoracic surgery; *n* = number of cases; and *N* = total number of cases in the literature review.

**Table 3:** Reported cases utilizing mediastinoscopy for diagnostic or therapeutic management.

Year/Reference	Specific Local Procedure	Tumor Size
2002/Kim et al. [21]	Mediastinoscopy + partial sternotomy (biopsy)	Not reported
2006/Park et al. [22]	Diagnostic mediastinoscopy (n=3)	Not reported
2014/Sadrizadeh et al. [23]	Video-assisted mediastinoscopy	Not reported
2025/Kuzucuoglu et al. [24]	Mediastinoscopy + excision	2.1 × 1.2 cm

**Table 4:** Non-surgical and conservative therapeutic strategies reported for adult mediastinal lymphangioma.

Year/Reference	Therapeutic Strategy and Clinical Rationale
2002 Yoo et al. [25]	Diagnostic fluid aspiration and an incisional biopsy, followed by external radiation therapy. Resection was not performed due to the systemic nature of his underlying disease (Gorham’s Disease).
2005 Coffing et al. [7]	FNA (fine-needle aspiration) of an asymptomatic 5.2 cm posterior mediastinal LM, in a patient with aggressive ovarian carcinoma
2011 Conte et al. [26]	Monitoring for an asymptomatic LM diagnosed 20 years prior
2012 Choi et al. [27]	EBUS aspiration of a large asymptomatic LM
2015 Golinelli et al. [28]	US-guided percutaneous sclerotherapy using OK-432 (Picibanil) for a 10 × 13 cm LM t near critical neurovascular bundles and vital structures
2017 Rali et al. [29]	Decompressive hemilaminectomies (C4–T5) to relieve spinal cord compression caused by a massive posterior mediastinal LM in a high-risk candidate.
2018 Malhotra et al. [30]	Emergency pericardial drainage and an endolymphatic stent graft (Viabahn endoprosthesis) across the thoracic duct in a LM previously treated with mTOR (Mammalian Target of Rapamycin) inhibitors.
2018 Nasser et al. [31]	Creation of a pericardial window followed by long-term mTOR inhibitor therapy (sirolimus) for an infiltrative mass encasing the aortopulmonary window.

## Data Availability

The original contributions presented in this study are included in the article. Further inquiries can be directed to the corresponding author.

## Abbreviations

CD31	Cluster of differentiation 31
CD34	Cluster of differentiation 34
CT	Computed tomography
DLCO	Diffusing capacity of the lung for carbon monoxide
EBUS	Endobronchial ultrasound
ERG	ETS-related gene
FNA	Fine-needle aspiration
HE	Hematoxylin and eosin
IQR	Interquartile range
KCO	Carbon monoxide transfer coefficient
LM	Mediastinal lymphangioma
MRI	Magnetic resonance imaging
mTOR	Mammalian target of rapamycin
N/R	Not reported
PET-CT	Positron emission tomography–computed tomography
PIK3CA	Phosphatidylinositol-4,5-bisphosphate 3-kinase catalytic subunit alpha
RATS	Robotic-assisted thoracic surgery
SD	Standard deviation
SMA	Smooth muscle actin
US	Ultrasonography
VATS	Video-assisted thoracic surgery
VEGF	Vascular endothelial growth factor

## Appendix A

**Table A1:** Chronological literature review of reported adult primary mediastinal lymphangioma cases (2001–2026).

Year	Author	No Cases	Sex	Age (Years) *	Clinical Presentation	Imaging	Size (CM)	Positioning in the Mediastinum	Surgical Approach	Histopathology
2001	Oshikiri et al. [50]	5	2M 3F	20–74	4 Asymptomatic, 1 dyspnea and superior vena cava syndrome	5CT 1MRI	11	1 Anterior 1 Superior 3 Posterior	N/R	3 cystic LM 1 cavernous LM 1 mixed LM
2002	Kim et al. [21]	1	M	77	Cough	CT +MRI	N/R	Anterior mediastinum	Mediastinoscopy + partial sternotomy + biopsy	Cavernous LM
2002	Yoo et al. [25]	1	M	38	Sudden onset of anterior chest and neck pain	CT	N/R	Anterior mediastinum	N/R	Cavernous LM
2003	Kim et al. [38]	3	F	48	Asymptomatic	CT	N/R	Superior, anterior, middle, and posterior mediastinum	N/R	Cystic LM
			M	31	Asymptomatic	CT + MRI	4 × 4	Anterior mediastinum	VATS	Cystic LM
			M	30	Asymptomatic	CT + MRI	13 × 10.5 × 0.2	Middle mediastinum	Thoracotomy	Cystic LM
2003	Shetty et al. [51]	1	M	43	Gradual dysphagia and significant weight loss	CT	15 × 10 × 7	Middle mediastinum	Right posterolateral thoracotomy	Cystic LM
2003	Zahirifard et al. [52]	1	M	28	1.5-month history of dyspnea on exertion and palpitations, with worsening dyspnea in the two weeks prior to admission	CT	N/R	All mediastinum compartments	Right posterolateral thoracotomy.	Cavernous LM
2004	Daya et al. [53]	1	M	45	Acute chest pain + intermittent episodes 2 weeks prior	CT	12 × 10	Anterior mediastinum	Median sternotomy	Cystic LM
2004	Parker et al. [39]	1	M	47	Asymptomatic	CT	15 × 7	Posterior mediastinum	VATS biopsy	Esophageal LM
2005	Coffing et al. [7]	1	F	46	Incidental finding	CT	5.2	Posterior mediastinum	None	
2005	Pop et al. [54]	1	M	60	Cardiac tamponade initially, 1 year later palpitations, dyspnea, tachycardia and pleural effusion	CT	9 × 5.5	Anterior mediastinum	Bilateral VATS	Cystic LM
2006	Misthos et al. [55]	4	4M	23–54 (mean 38.7)	Asymptomatic	CT	7–13 (mean 9.5)	Superior mediastinum	Muscle-sparing lateral minithoracotomy	Cystic LM
2006	Park et al. [22]	8	F	48	Chest pain, dyspnea	CT	N/R	Right superior and middle mediastinum	Primary: Thoracotomy (performed in 6 cases as the initial procedure). Diagnostic/Secondary: Mediastinoscopy (3 patients) and Mediastinotomy (3 patients) were used to obtain diagnosis or for patients with smaller/accessible paratracheal lesions	Cavernous or cystic LM
			M	66	Dyspnea	CT	N/R	Right and middle mediastinum		
			F	81	Dyspnea	CT	N/R	Right superior and middle mediastinum		
			F	26	Chest pain, dyspnea, hemoptysis	CT	N/R	Superior mediastinum		
			F	41	Asymptomatic	CT	N/R	Right superior and posterior mediastinum		
			F	70	Chest pain, dyspnea, hemoptysis	CT	N/R	Right anterior mediastinum		
			F	39	Dyspnea	CT	4	Right superior mediastinum		
			M	47	Fever	CT	7 × 8 × 5	Right superior mediastinum		
2006	Saleiro et al. [56]	1	F	44	Asymptomatic	CT	4 × 5	Anterior and superior mediastinum	Right posterolateral thoracotomy	Cystic LM
2008	Correia et al. [5]	1	M	50	Incidental finding on X-ray	CT	4.5	Superior mediastinum	Right lateral thoracotomy	Cystic LM
2008	Teramoto et al. [57]	1	F	43	One-month history of cough, dyspnea, and recurrent high fevers (up to 40 °C)	CT + MRI	3.8 × 3 × 7.5	Posterior mediastinum	VATS converted to right thoracotomy	Cavernous LM
2009	Hunt et al. [40]	1	F	43	Asymptomatic	CT	N/R	Superior mediastinum	VATS	Cystic LM
2009	Melo et al. [58]	1	F	59	Asymptomatic	CT	4.5	Mediastinum right paratracheal	Right lateral thoracotomy	Cystic LM
2009	Sun yi-feng et al. [59]	7	3M 4F	18–67 (average 40)	Asymptomatic	CT	5–8	Superior mediastinum	Median sternotomy (4 patients), right posterolateral thoracotomy (1 patient), cervical supraclavicular incision (2 patients)	Cystic LM
2011	Conte et al. [26]	1	F	60	Palpitations	MRI	2.5 × 7	Located between the visceral and parietal pericardium; attached to the heart but not infiltrating the ventricular wall	N/R	Cystic LM
2011	Kanzaki et al. [41]	1	F	20	Palpitation, cough, and dyspnea	CT + MRI	13 × 10	Anterior mediastinum	Bilateral VATS	Cystic LM
2012	Choi et al. [27]	1	M	29	Dry cough	CT + MRI	13.7	N/R	N/R	Cystic LM
2014	Mizutani et al. [60]	1	M	38	Asymptomatic	CT + MRI	7	Left anterior mediastinum	VATS	Cystic LM
2014	Fokkema et al. [32]	1	M	29	Dysphagia	CT	N/R	Anterior and middle mediastinum	Partial sternotomy/Hemiclamshell thoracotomy	N/R
2014	Komatsu et al. [61]	1	M	70	Dyspnea on exertion	CT + MRI	8	Superior mediastinum	VATS	Cystic LM
2014	Lazopoulos et al. [42]	1	M	30	Asymptomatic	CT + MRI	19 × 9.8 × 16.3	Anterior mediastinum	Right VATS	Cystic LM
2014	Sadrizadeh et al. [23]	1	M	80	Cough and rest dyspnea	CT	N/R	Superior and middle mediastinum	Video-assisted mediastinoscopy	Cystic LM
2014	Yang et al. [62]	1	M	46	Asymptomatic	CT	18	Right anterior mediastinum	VATS	Cystic LM
2015	Golinelli et al. [28]	1	F	43	Right-side supraclavicular mass (present for ~1 year) with accelerated growth in recent months. Painless and soft-elastic consistency	US, Doppler US and MRI	10 × 13	Anterior and superior mediastinum	N/R	Cystic LM
2015	Lee et al. [63]	1	F	66	Asymptomatic	CT	3.4	Right upper anterior mediastinum	VATS	Cystic LM
2015	Suehisa et al. [13]	1	M	35	Asymptomatic	CT + MRI	7.5	Left anterior Mediastinum	left VATS	Cystic LM
2016	Swarnakar et al. [43]	1	M	36	One-year history of dyspnea on exertion; one-month history of low-grade fever	CT	N/R	Middle mediastinum	VATS	Cystic LM
2017	Rali et al. [29]	1	M	23	Chronic dyspnea and wheezing; 2-month history of progressive extremity weakness, paresthesia, and numbness	Ct + MRI	17 × 16 × 12	Middle and posterior mediastinum	N/R	Cystic LM
2017	Zhou et al. [14]	1	F	69	Chest pain for 1 month	CT	10.5 × 7.5	Posterior mediastinum	Right VATS	Cystic LM
2018	Malhotra et al. [30]	1	F	21	Initially: Pleuritic chest pain and night sweats. 8 months later: Pericardial tamponade and cardiac arrest	CT + lymphangiography	9 × 5.3	Anterior mediastinum	N/R	N/R
2018	Nasser et al. [31]	1	F	32	Subacute onset of fever, shortness of breath, and fatigue; presented with decompensated cardiac tamponade and hemodynamic instability	US + MRI	13 × 11.8	Middle mediastinum	N/R	N/R
2019	Dionisio et al. [35]	1	F	36	Presented to ER for unrelated weakness and vomiting. Denied chest pain, dyspnea, cough, or fever	CT +MRI	20 × 13 × 14	Medial and anterior mediastinum	N/R	Cystic LM
2019	Dirol et al. [64]	1	M	46	Asymptomatic	CT	7.5 × 4.5	Anterior and superior mediastinum	Right lateral thoracotomy	Cystic LM
2019	Salehi et al. [37]	1	F	18	Left-sided chest pain and worsening shortness of breath	CT + US	N/R	Left middle mediastinum	VATS to thoracotomy	Cystic LM
2021	Reddy et al. [36]	1	F	27	4-day history of upper abdominal discomfort and breathing difficulty in the left lateral position; presented with impending cardiac tamponade	US	N/R	Middle mediastinum	Right VATS	Cystic LM
2021	Youssef et al. [65]	1	M	44	Incidental discovery on X-ray; however, patient retrospectively reported 6 months of cough, dyspnea on exertion, and shoulder pain	CT	6.5 × 3.5 × 4	Superior mediastinum	Right posterolateral thoracotomy	Cystic LM
2022	Li et al. [48]	1	F	50	Acute onset of left-sided pleuritic chest pain	CT + MRI	13.2	Mediastinum and entire left hemithorax	Emergency thoracotomy	Cystic LM
2024	Quaher et al. [8]	1	F	35	Persistent, dull, non-radiating central chest pain for two months	CT + MRI + PET-CT	6.5 × 4.2 × 5	Anterior and superior mediastinum	VATS	Cystic LM
2025	Akiba et al. [66]	1	F	76	Asymptomatic	CT + MRI	7	Anterior mediastinum	VATS	Cystic LM
2025	Kuzucuoğlu et al. [24]	1	M	61	Hoarseness	CT + PET-CT	2 × 1.2	Superior mediastinum	Mediastinoscopy + excision	Cystic LM
2026	Al Mahrizi et al. [33]	1	M	24	Chest pain, cough, intermittent fever, night sweats, decreased appetite	CT	7.5 × 5.2 × 6.8	Anterior mediastinum	Robotic-assisted thoracoscopic resection	N/R

* for papers with more than one case, the number represents either an average or interval, as offered by the article. M—male, F—female.
